# Editorial: New Insights and Updates on the Molecular Epidemiology and Antimicrobial Resistance of MRSA in Humans in the Whole-Genome Sequencing Era

**DOI:** 10.3389/fmicb.2019.00637

**Published:** 2019-04-03

**Authors:** David C. Coleman, Anna C. Shore, Richard V. Goering, Stefan Monecke

**Affiliations:** ^1^Microbiology Research Unit, Division of Oral Biosciences, Dublin Dental University Hospital, University of Dublin, Trinity College Dublin, Dublin, Ireland; ^2^Department of Medical Microbiology and Immunology, School of Medicine, Creighton University, Omaha, NE, United States; ^3^Leibniz Institute of Photonic Technology (IPHT), InfectoGnostics Research Campus, Jena, Germany; ^4^Institute for Medical Microbiology and Hygiene, Medical Faculty “Carl Gustav Carus”, Technische Universität Dresden, Dresden, Germany

**Keywords:** MRSA, whole-genome sequencing, evolution, molecular epidemiology, antimicrobial resistance

Methicillin resistant *Staphylococcus aureus* (MRSA) have been a significant cause of healthcare-associated (HA) infection globally for several decades and more recently, community-associated (CA) infection. Surveillance of MRSA locally, nationally and internationally is critical in monitoring its emergence and spread and informing prevention strategies. Molecular typing has played a key role in tracking the spread of MRSA for many years using a variety of DNA-based methods such as pulsed-field gel electrophoresis typing, *spa* typing and conventional multilocus sequence typing (MLST). However, these approaches are often inadequately discriminatory. The advent of high-throughput whole-genome sequencing (WGS) has revolutionized MRSA research by enabling the complete genomic sequence of isolates to be determined and compared. This has facilitated enhanced outbreak detection and has provided in-depth insights into the evolution of particular MRSA clones and the emergence of new clones.

The objective of this topic was to assemble a range of articles on important aspects of HA- and CA-MRSA in humans, informed by WGS technology. The research topic consists of seven original research articles and two descriptive reviews which focus on up-to-date perspectives in the areas of molecular epidemiology and spread of MRSA and highlights how WGS has revolutionized the epidemiological and phylogenetic investigation of specific MRSA clones in different parts of the world. The topic also highlights the impact of WGS on our understanding of antimicrobial resistance encoded by mobile genetic elements (MGEs) including staphylococcal cassette chromosome *mec* (SCC*mec*) elements and the role of specific coagulase-negative staphylococcal species (CoNS) in the evolution of MGEs, such as the arginine catabolic mobile element (ACME) which can enhance the survival of MRSA following acquisition.

Two of the studies highlight the usefulness and accuracy of WGS, using different analysis methods, for protracted outbreak investigations of two MRSA clones uncommon in the regions in question.

Earls et al. used WGS to investigate the relatedness of CA clonal complex (CC) 88-MRSA isolates from two separate protracted outbreaks (2009–2011 and 2014–2017) in the neonatal intensive care unit of an Irish hospital and their likely geographic origin. Outbreak I included seven *spa* type t186 isolates from seven patients, whereas outbreak II consisted of 15 *spa* type t786 isolates including 13 patient isolates and two isolates from the same healthcare worker (HCW) recovered 2 years apart. All isolates were identified as sequence type (ST) 78-MRSA-IVa and formed a large cluster in a whole-genome MLST-based minimum spanning tree and exhibited 1-71 pairwise allelic differences. Interestingly, in addition to having a different *spa* type, all outbreak II isolates lacked the *hsdS* gene. The two HCW isolates exhibited one allelic difference despite being recovered 2 years apart. Core-genome MLST and sequence-based plasmid analysis revealed the recent shared ancestry of Irish and Australian ST78-MRSA-IVa. The results of this study revealed that the ST78-MRSA-IVa from both protracted outbreaks formed a homogenous population despite differences in *spa* type and the presence/absence of the *hsdS* gene.

Rubin et al. used WGS to investigate a protracted outbreak of ST97-MRSA-IVa over a 4-year period in a surgical ward in a hospital in Denmark involving 23 patients and two HCWs. Eighteen of the patients had been admitted to the surgical ward, one had shared a ward at another hospital with a patient previously admitted to the same surgical ward and two were family members of MRSA-positive patients. Twelve of the patients were diagnosed with ST97-MRSA-IV in the community. The 25 ST97-MRSA-IVa isolates were remarkably homogenous exhibiting a maximum of 50 single nucleotide polymorphisms (SNPs) for isolates recovered over a 4-year period. A subset of the 15 isolates recovered from 13 patients admitted during 2016 and 2017 and the two HCW isolates exhibited a maximum of 15 SNPs. The authors hypothesized that colonized HCWs may have contributed to the maintenance of the protracted outbreak. Both of these studies highlight how WGS can enhance the detection and analysis of prolonged hospital outbreaks of MRSA and that HCWs with an unknown MRSA carriers state might cause sporadic transmission and sustain an outbreak over several years.

Three of the studies undertook large-scale investigations of the evolution of some key successful MRSA lineages over several decades.

van Hal et al. used WGS to examine 459 ST93 *S. aureus* from Australia, New Zealand, Samoa, and Europe to explore the evolution of ST93, its emergence in Australia and subsequent spread to other countries. In Australia, Panton–Valentine leukocidin-positive ST93-MRSA has rapidly become the most common CA-MRSA lineage. Comparisons with other *S. aureus* genomes indicated that ST93 is an early diverging and recombinant lineage, encompassing segments from the ST59/ST121 lineage. Limited genetic diversity was observed within extant ST93 with the most recent common ancestor dated to 1977. The analysis revealed that an epidemic ST93 population arose from a methicillin-susceptible *S. aureus* (MSSA) ancestor in remote Northern Australia, which has a proportionally large population of native Australians with a high prevalence of skin and soft tissue infections. The analysis further revealed that methicillin resistance was acquired by ST93 three times in these regions with an ST93-MRSA-IVa clade expanding and spreading to Australia's east coast by 2000. Non-sustained introductions of ST93-MRSA-IVa to the United Kingdom were observed, whereas ST93-MRSA-IVa was sustainably transmitted with clonal expansion within the Pacific Islander population in New Zealand who experience similar socio-economic disadvantages as native Australians. This study highlights the significance of human population factors in the emergence of CA-MRSA.

Challagundla et al. undertook the first large-scale phylogenomic study of CC5-MRSA from the Western Hemisphere in order to elucidate the phylogeny of major clones, their place and time of origin and to map the evolution of associated key characteristics. MRSA clones belonging to CC5 are highly diverse and have been a major cause of HA infection in the Western Hemisphere for decades. The study investigated 598 genome sequences, including 409 newly generated sequences, which identified a geographically well-dispersed early branching CC5-Basal clade consisting of MSSA and MRSA. The basal clade gave rise to two major clades in the early 1960's and early 1970's that underwent major expansions in the Western Hemisphere including a CC5-I clade in South America and a CC5-II clade largely in Central and North America. The clade expansions were preceded by convergent acquisitions of resistance to several antibiotic classes and convergent losses of the *sep* gene encoding staphylococcal enterotoxin p. Unique losses of surface proteins were also noted for both clades. The study also revealed that the recombination rate of CC5 was much lower than previously reported for other *S. aureus* lineages. Overall, the results of this study elucidated the relationships between CC5 clades and clones and identified genomic changes for increased antibiotic resistance and decreased virulence associated with CC5 MRSA clade expansions in the Western Hemisphere. These findings suggest that less virulent and more antibiotic resistant CC5-MRSA clones may be better adapted to spread geographically.

Monecke et al. investigated ST239-MRSA-III, a pandemic strain circulating in many countries globally since the 1970s, which has largely been displaced by other MRSA strains in Europe in recent decades. A total of 184 isolates from 11 different countries were characterized using DNA microarrays that profiled an extensive range of typing markers, virulence and antimicrobial agent resistance genes and SCC*mec* types. Thirty additional isolates were subjected to WGS and, together with published WGS data for 215 additional ST239-MRSA-III isolates, were analyzed *in-silico* for comparison with the microarray. This approach assigned the isolates to 39 different SCC*mec* III subtypes, and to three major and several minor clades. One clade comprising isolates and sequences from Turkey, Romania and other Eastern European countries, Russia, Pakistan, and Northern China was characterized by the integration of a transposon into the *nsaB* gene and by the loss of the *fnbB* and s*plE* genes. Another clade, harboring s*asX*/*sesI* was found to be widespread in South-East Asia including China/Hong Kong, and in Trinidad & Tobago. A third, related, but s*asX*/*sesI*-negative clade occurs in Latin America but also in Russia and in the Middle East from where it apparently originated. This study demonstrated that for pandemic ST239-MRSA-III, analysis of genome markers assigned by array hybridization, multiplex PCR, or by WGS can help assigning clinical isolates to these clades or variants and thus help to identify the likely provenance of an isolate.

Aside from providing unparalleled high-resolution for epidemiological, evolutionary and population structure investigations, WGS applications have also enabled the direct comparison of large, distinctive genomic regions associated with pathogenesis or antimicrobial resistance, amongst distinct isolates and species.

McClure et al. applied WGS to the genomic comparison of four closely related ST239 MRSA strains; one avirulent, two moderately virulent and one virulent, in order to gain insights into which areas of the genome might contribute to enhanced pathogenicity. The results revealed that the most virulent strain harbored the complete and intact *spa* and *lpl* genes, both of which contribute to innate immunity and virulence. In the moderately virulent strains, the *spa* gene was present and identical whereas the *lpl* gene was disrupted. Both the *spa* and *lpl* genes were absent in the avirulent strain. The authors also implicated MGEs encoding virulence and antibiotic resistance determinants in strain pathogenicity differences. The MGE arsenal was largest in the virulent strain, although several similar MGEs were also detected in the other three less virulent strains. This study highlights the sensitive interplay that exists between distinct virulence factors in the overall pathogenicity of a strain.

Although first described as a MGE contributing to the growth and survival capability of the USA300-MRSA clone, the high prevalence and extensive diversity of ACME amongst *S. epidermidis* was revealed by O'Connor et al. using WGS. Five main ACME types and further subtypes ranging between 27 and 117 kb were described in oral *S. epidermidis* isolates recovered from the oral rinse, periodontal pockets and subgingival sites of orally healthy individuals with and without dental implants, and in patients with periodontal disease or infected dental implants. This study suggested that ACME may contribute to the survival of *S. epidermidis* in oral environments, likely due to the contribution of the *arc* and *kdp* operons (encoding an arginine deaminase pathway and potassium transporter, respectively) harbored by ACME types I, II, IV, and V. This study also highlighted the role of CoNS in the evolution of *S. aureus* and MRSA, due to its ability to act as a reservoir for MGEs that contribute to strain fitness and survival.

The review by Miragaia outlines the intricate combination of distinct factors that contributed to the emergence, spread and evolution of MRSA, in addition to the application of WGS in deciphering these individual contributory factors. This article describes the pre-existence of native *mec* genes harbored by staphylococcal species predominantly associated with environmental- and animal-sources, the stepwise assembly and diversification of staphylococcal cassette chromosome incorporating *mec* (SCC*mec*) elements in other CoNS and subsequent transfer to *S. aureus*. The historical context of clinical treatment and farming practices are discussed in relation to the emergence of MRSA as one of the most important worldwide pandemics, originating in hospitals before spreading into the community and livestock.

Lastly, the mini-review by van Belkum and Rochas provides a succinct overview of current laboratory-based and point-of-care testing for MSSA/MRSA detection. In recent years there has been a significant move from culture-based, phenotypic methods toward molecular detection due to the strong correlation between phenotypic resistance and the presence of *mec* genes encoding methicillin resistance. The review highlights the range of approaches, mostly polymerase chain reaction-based detection methods, used for molecular detection of MRSA/MSSA in the laboratory setting or at the point-of-care. The potential diagnostic impact of WGS technology is discussed against a background of diagnostic, surveillance, and infection control parameters and toward enabling rapid pathogen detection and determination of virulence characteristics and antibiotic resistance profiles, potentially directly from clinical specimens.

These articles and reviews emphasize the significant impact that WGS has had on our understanding of the surveillance, epidemiology, and evolution of MRSA and factors that drive the emergence and spread of specific clones. They also reveal the enormous potential impact WGS technology will have on the future detection, prevention, and treatment of MRSA.

## Author Contributions

DC, AS, RG, and SM conceived the research topic, recruited the contributing authors and edited the topic manuscripts. DC drafted the editorial. AS, RG, and SM reviewed and edited the draft editorial. All authors approved the final editorial.

### Conflict of Interest Statement

The authors declare that the research was conducted in the absence of any commercial or financial relationships that could be construed as a potential conflict of interest.

